# Sublobar resection versus ablation for stage I non-small-cell lung cancer: a meta-analysis

**DOI:** 10.1186/s13019-022-01766-1

**Published:** 2022-02-11

**Authors:** Yong Li, Fang Yang, Ya-Yong Huang, Tao Wang

**Affiliations:** 1 Sichuan Key Laboratory of Medical Imaging and Department of Radiology, Affiliated Hospital of North Sichuan Medical College, Nanchong, China; 2 Department of Radiology, Xuzhou Central Hospital, 199 South Jiefang Road, Xuzhou, China

**Keywords:** Sublobar resection, Ablation, Lung cancer, Meta-analysis

## Abstract

**Background:**

Stage I non-small-cell lung cancer (NSCLC) can be treated by both ablation and sublobar resection (SR). This meta-analysis was therefore designed to better compare the relative safety and efficacy of these two approaches to treating stage I NSCLC.

**Materials and methods:**

Relevant studies published through November 2020 in the Cochrane Library, Embase, and PubMed databases were identified for analyses which were conducted with RevMan v5.3.

**Results:**

In total, 816 potentially relevant articles were identified, of which 8 were ultimately included in the final meta-analysis. Patients in the SR group exhibited a signficantly lower pooled local recurrence (LR) rate (5.0% vs. 25.4%, *P* < 0.0001), although pooled distant recurrence (DR) rates were similar in both groups (25.7% vs. 23.1%, *P* = 0.75). The pooled hazard ratio (HR) for overall survival (OS) (HR: 1.23; 95% CI: 1.13–1.33, *P* < 0.00001), progression-free survival (PFS) (HR: 1.34; 95% CI: 1.15–1.55, *P* = 0.0002), and cancer-specific survival (HR: 1.39; 95% CI: 1.15–1.70, *P* = 0.0009) all indicated better survival outcomes among patients that underwent HR treatment, while pooled complication rates were similar in both groups (27.7% vs. 43.8%, *P* = 0.27). Patients that underwent ablation exhibited significantly shorter pooled post-operative hospitalization relative to those in the SR group (MD: 5.93; 95% CI: 0.78–11.07, *P* = 0.02). No evidence of publication bias was detected through funnel plot analyses.

**Conclusions:**

SR treatment of stage I NSCLC patients was associated with a lower LR rate and longer survival as compared to ablation.

## Background

Non-small-cell lung cancer (NSCLC) is the deadliest form of cancer globally [1–3], and many patients with early-stage NSCLC can undergo curative surgical resection following tumor detection via chest computed tomography (CT) screening [4–6]. Lobectomy with systematic mediastinal lymphadenectomy is the standard approach to treating stage I NSCLC [7–9]. However, in many cases, patients are elderly or have a history of comorbidities such as pulmonary dysfunction or atherosclerosis that may preclude their ability to undergo invasive surgical treatment s [4–6].

While sublobar resection (SR) is generally less effective as a curative procedure relative to lobectomy [4], it can better preserve patient pulmonary function [7–9]. In stage I NSCLC patients with tumors ≤ 2 cm in size, SR yields similar survival outcomes to lobectomy [5, 6]. However, 20–30% of stage I NSCLC patients are unable to undergo SR owing to their poor performance status [10–17]. In these patients, percutaneous ablation represents the most minimally invasive treatment strategy [10–17]. While prior studies have compared the relative efficacy of SR and ablation in stage I NSCLC patients, the majority of these analyses have been retrospective in design [10–17]. Conducting a meta-analysis would thus represent an effective means of reducing potential bias and increasing statistical power associated in order to develop more reliable conclusions.

The present meta-analysis was therefore designed to compare the relative safety and efficacy of SR and ablation for the treatment of individuals with stage I NSCLC.

## Methods

### Study selection

This meta-analysis was conducted in accordance with the Preferred Reporting Items for Systematic reviews and Meta-Analyses (PRISMA) statement. This work was registered in https://inplasy.com/ (No. INPLASY202110075).

Relevant studies published as of November 2020 in the Cochrane Library, Embase, and PubMed databases were identified via the following search strategy: (((((radiofrequency[Title/Abstract]) OR (microwave[Title/Abstract])) OR (cryoablation[Title/Abstract])) OR (ablation[Title/Abstract])) AND ((((surgery[Title/Abstract]) OR (resection[Title/Abstract])) OR (Video assisted thoracoscopic surgery[Title/Abstract])) OR (VATS[Title/Abstract]))) AND ((lung cancer[Title/Abstract]) OR (NSCLC[Title/Abstract])).

Studies eligible for inclusion in this meta-analysis included (a) randomized controlled trials (RCTs) or retrospective analyses, (b) studies of stage I NSCLC patients, (c) studies in which SR and ablation were compared to treat NSCLC. Studies published in any language were eligible for inclusion.

Studies were excluded if they were (a) non-comparative studies, (b) animal or preclinical studies, or (c) reviews.

### Data extraction

Baseline patient data, baseline study data, and treatment-related data were independently extracted by two investigators. Discussion with a third author was used to resolve any inconsistencies.

### Quality assessment

Study quality was assessed independently by two researchers. RCT quality was assessed with the Cochrane risk of bias tool based upon selection, performance, detection, attrition, reporting, and other biases. All other studies were evaluated using the 9-point Newcastle–Ottawa scale (NOS) [18], with high-quality studies being those scoring ≥ 6 points.

### Endpoints and definitions

Meta-analysis endpoints included rates of complications, recurrence rates, duration of postoperative hospitalization, and patient survival, with survival as the primary study endpoint.

For this study, SR was defined as both segmental and wedge resection operations [10–17]. Both local recurrence (LR) and distant recurrence (DR) outcomes were evaluated, with the latter of these including intra- and extra-pulmonary DR [11, 12]. Analyzed survival outcomes included overall, progression-free, and cancer-specific survival (OS, PFS, and CSS, respectively).

### Meta-analysis

RevMan v5.3 was used for all meta-analyses. Pooled odds ratios (ORs) and corresponding 95% confidence intervals (CIs) were calculated via the Mantel–Haenszel method for dichotomous variables, whereas continuous variables were analyzed based upon mean difference (MD) values and 95% CIs. Pooled survival duration was assessed based upon hazard ratios (HRs) and 95% CIs. Study heterogeneity was evaluated based upon X^2^ tests and the I^2^ statistic, with I^2^ > 50% being indicative of significant heterogeneity. When significant heterogeneity was detected, data were analyzed with a random-effects model, whereas fixed-effects models were otherwise utilized. Sources of heterogeneity were assessed with sensitivity and subgroup analyses, and funnel plots were used to test the risk of publication bias.

## Results

### Study characteristics

**Fig. 1:**
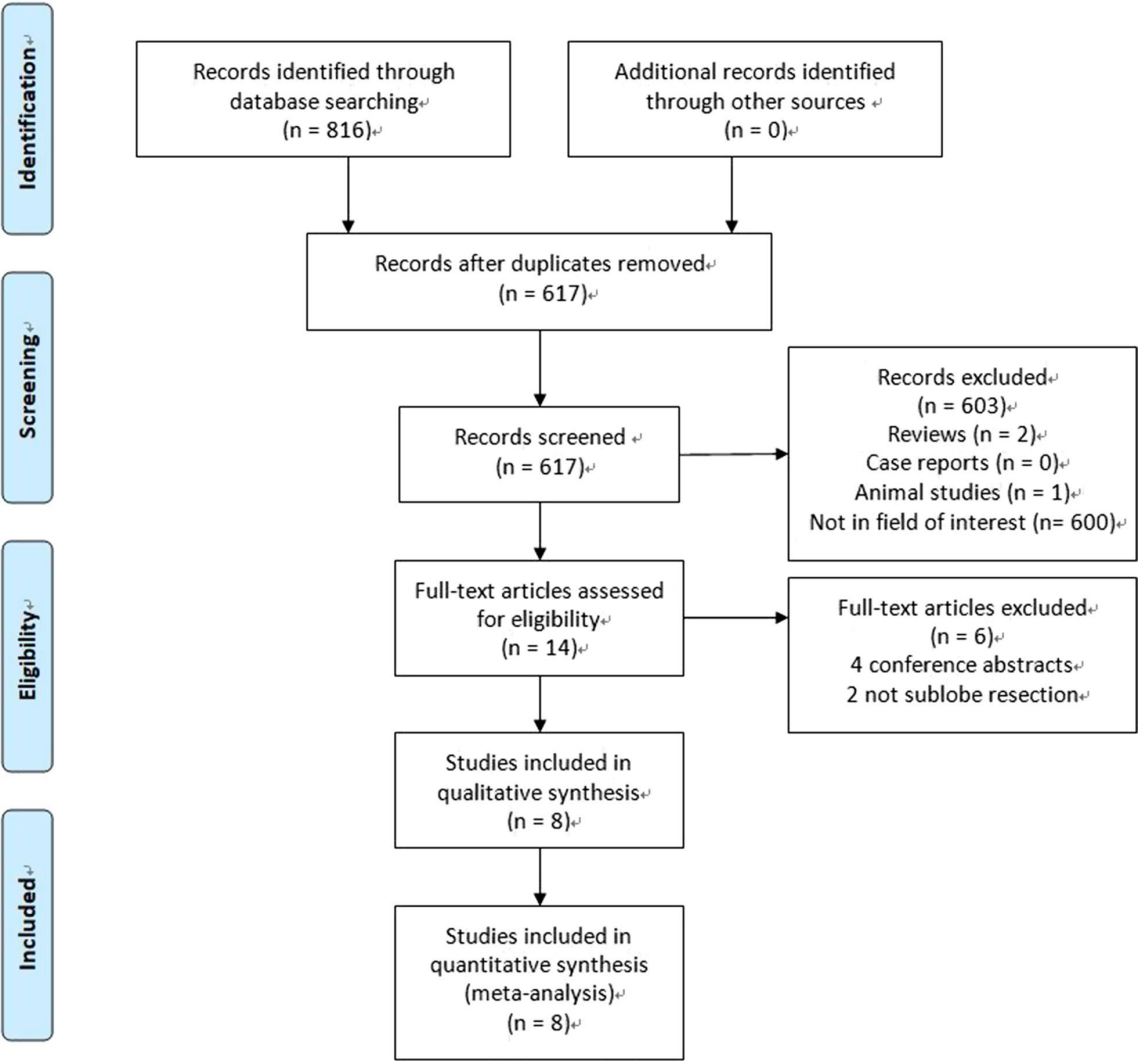
Flowchart diagram of our meta-analysis

The initial search strategy identified 816 potentially relevant articles, of which eight were ultimately included in our final meta-analysis (Fig. 1). These were all retrospective studies with NOS scores between 6 and 8 points (Table 1), incorporating 679 patients treated by SR and 468 that underwent ablation (Tables 1, 2). In five studies, patients underwent radiofrequency ablation (RFA) [10, 11, 13, 15, 16], while in three studies, wedge resection and ablation treatments were compared [11, 12, 17]. Treatment-associated data for these studies are shown in Table 2.

**Table 1:** Characteristics of the included studies

Study/year/country	Groups	Sample size	Male/female	Age (years)	Tumor size (mm)	FEV1/predicted (%)	CCMI	NOS
Alexander/2013/USA [10]	SR	28	12/16	74	NG	54	42.61	7
	Ablation	56	24/32	78	NG	52	42.45	
Ambrogi/2015/Italy [11]	SR	59	46/13	70	26	47	NG	8
	Ablation	62	45/17	76	23	49	NG	
Hu/2020/China [12]	SR	155	103/52	78	25	71	4^a^	8
	Ablation	68	44/24	83	23	63	4^a^	
Iguchi/2020/Japan [13]	SR	193	101/92	67	16	NG	2.78^a^	7
	Ablation	38	22/16	75	22	NG	3.03^a^	
Kwan/2014/USA [14]	SR	69	36/33	NG	NG	NG	NG	6
	Ablation	99	26/73	NG	NG	NG	NG	
Safi/2015/Germany [15]	SR	42	27/15	70	19	69	6.2	8
	Ablation	25	18/7	71	22	67	5.5	
Zemlyak/2010/USA [16]	SR	25	9/16	66	NG	65	NG	7
	Ablation	12	7/5	74	NG	64	NG	
Zeng/2020/China [17]	SR	108	58/60	70.2	NG	NG	NG	7
	Ablation	108	44/64	68.1	NG	NG	NG	

*CCMI* Charlson Comorbidity Index, *SR* sublobar resection, *NG* not given, *NOS* Newcastle–Ottawa scale

^a^The Charlson comorbidity index was significant lower in SR group

**Table 2:** Characteristics of the treatment outcomes

Study	Groups	SR methods	Ablation	LR (%)	DR (%)	Complication (%)	Hospital stay
Alexande [10]	SR	Segmental and wedge	RFA	Not given	Not given	53.6%	Not given
	Ablation			Not given	Not given	62.3%	Not given
Ambrogi [11]	SR	Wedge	RFA	1.7%	11.9%	27.1%	6 days
	Ablation			22.6%	11.3%	21.0%	2 days
Hu [12]	SR	Wedge	Microwave	5.2%	31.0%	Not given	Not given
	Ablation			26.5%	33.8%	Not given	Not given
Iguchi [13]	SR	Segmental and wedge	RFA	Not given	Not given	368 days	16 days
	Ablation			Not given	Not given	222 days	6.5 days
Kwan [14]	SR	Segmental and wedge	No details	Not given	Not given	232 days	Not given
	Ablation			Not given	Not given	110 days	Not given
Safi [15]	SR	Segmental and wedge	RFA	Not given	Not given	9.8 months	Not given
	Ablation			Not given	Not given	5.6 months	Not given
Zemlyak [16]	SR	Segmental and wedge	RFA	12%	Not given	0	Not given
	Ablation			33.3%	Not given	66.7%	Not given
Zeng [17]	SR	Wedge	No details	Not given	Not given	194 days	Not given
	Ablation			Not given	Not given	86 days	Not given

*LR* local recurrence, *DR* distant recurrence, *RFA* radiofrequency ablation, *SR* sublobar resection

### Recurrence

**Fig. 2:**
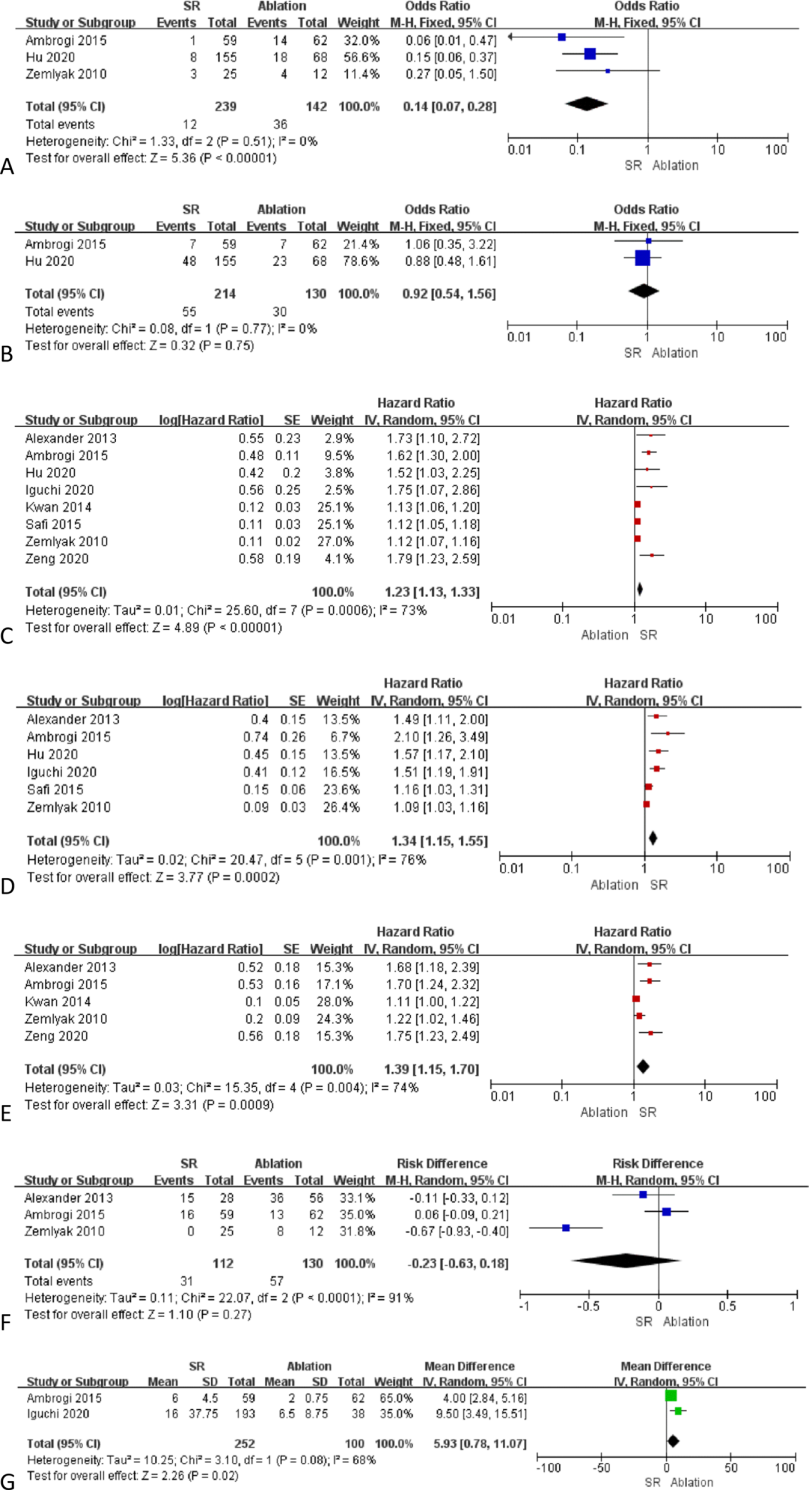
The pooled results of **A** LR rates, **B** DR rates, **C** OS, **D** PFS, **E** CSS, **F** complication rates, and **G** post-operative hospital stay via forest plots

LR was reported in three studies [11, 12, 16], with pooled LR rates being significantly lower in the SR group (5.0% vs. 25.4%, *P* < 0.0001, Fig. 2a). No significant heterogeneity pertaining to this endpoint was detected (I^2^ = 0%).

DR was reported in two studies [11, 12], and pooled DR rates were similar between groups (25.7% vs. 23.1%, *P* = 0.75, Fig. 2b). No significant heterogeneity pertaining to this endpoint was detected (I^2^ = 0%).

### Survival

OS was reported in all studies, and the pooled OS HR was more favorable in the SR group (HR: 1.23; 95% CI: 1.13–1.33, *P* < 0.00001, Fig. 2c). This endpoint was associated with significant heterogeneity (I^2^ = 73%), but omitting individual studies in a sensitivity analysis had no significant impact on overall heterogeneity.

PFS was reported in six studies [10–13, 15, 16], with the pooled PFS HR being more favorable in the SR group (HR: 1.34; 95% CI: 1.15–1.55, *P* = 0.0002, Fig. 2d). This endpoint was associated with significant heterogeneity (I^2^ = 76%), but omitting individual studies in a sensitivity analysis had no significant impact on overall heterogeneity.

CSS was reported in five studies [10, 11, 14, 16, 17], with the pooled CSS HR being more favorable in the SR group (HR: 1.39; 95% CI: 1.15–1.70, *P* = 0.0009, Fig. 2e). This endpoint was associated with significant heterogeneity (I^2^ = 74%), but omitting individual studies in a sensitivity analysis had no significant impact on overall heterogeneity.

### Complications

Complication rates were reported in three studies [10, 11, 16], and were similar in both groups (27.7% vs. 43.8%, *P* = 0.27, Fig. 2f). This endpoint was associated with significant heterogeneity (I^2^ = 91%), and the omission of the study conducted by Zemlyak et al.[16] eliminated this heterogeneity. When this study was omitted, pooled complication rates remained similar between groups (*P* = 0.98).

### Duration of postoperative hospitalization

Two studies reported the duration of postoperative hospitalization for treated patients [11, 13]. The pooled duration of postoperative hospitalization was significantly lower in the ablation group relative to the SR group (MD: 5.93; 95% CI: 0.78–11.07, *P* = 0.02, Fig. 2h). This endpoint was associated with significant heterogeneity (I^2^ = 68%).

### Subgroup analyses

Five articles specifically compared SR and RFA as approaches to treating stage I NSCLC [10, 11, 13, 15, 16], and in these studies, pooled HRs pertaining to OS, PFS, and CSS were all favorable in the SR group (Table 3).

**Table 3:** Meta-analytic pooled results of survival based on the RFA versus SR

	Number of studies	HR (95% CI)	Heterogeneity	Favor
Overall survival	5	1.24 (1.11, 1.38), *P* = 0.0002	*I*^2^ = 77%	SR
Progression-free survival	5	1.30 (1.11, 1.51), *P* = 0.001	*I*^2^ = 75%	SR
Cancer-specific survival	4	1.33 (1.09, 1.62), *P* = 0.005	*I*^2^ = 73%	SR

*HR* hazard ratio, *CI* confidential interval, *RFA* radiofrequency ablation, *SR* sublobar resection

Three articles specifically compared wedge resection and ablation as approaches to treating stage I NSCLC [11, 12, 17], and in these studies, pooled HRs pertaining to OS, PFS, and CSS were all favorable in the SR group (Table 4).

**Table 4:** Meta-analytic pooled results of survival based on the ablation versus wedge resection

	Number of studies	HR (95% CI)	Heterogeneity	Favor
Overall survival	3	1.63 (1.38, 1.93), *P* < 0.00001	*I*^2^ = 0%	SR
Progression-free survival	2	1.69 (1.31, 2.18), *P* < 0.00001	*I*^2^ = 0%	SR
Cancer-specific survival	2	1.72 (1.36, 2.18), *P* < 0.00001	*I*^2^ = 0%	SR

*HR* hazard ratio, *CI* confidential interval, *SR* sublobar resection

### Publication bias

No evidence of publication bias was detected through funnel plot analyses.

## Discussion

Current American College of Chest Physicians clinical practice guidelines suggest that stage I NSCLC patients undergo lobectomy with systematic mediastinal lymph node dissection when possible. SR is the preferred treatment in patients considered at high risk for lobar resection, although some studies have suggested that lobectomy and SR are associated with similar outcomes in those with stage I NSCLC [19, 20]. When patients are unable to tolerate SR, it is typically recommended that patients undergo ablation [14].

The present meta-analysis compared the relative safety and efficacy of SR and ablation for the treatment of stage I NSCLC. When comparing recurrence rates between these two patient groups, our analysis revealed LR rates to be significantly lower in the SR group (5.0% vs. 25.4%, *P* < 0.0001). This suggests that ablation cannot fully eliminate tumors, consistent with the fact that this approach is less effective when treating tumors adjacent to large vessels > 3 mm in diameter or to a bronchus > 2 mm in diameter owing to heat-sink effects [13]. However, there are certain advantages to the ablation procedure, including the fact that it can be performed repeatedly and can be employed to treat both synchronous and metachronous lesions [21]. We observed similar pooled DR rates in both patient groups (25.7% vs. 23.1%, *P* = 0.75), indicating that both SR and ablation are limited in their ability to control systematic tumor growth. Postoperative chemotherapy may represent a viable means of lowering DR rates [22].

Stage I NSCLC patient treatment primarily focuses on improving patient survival rates. We therefore analyzed OS, PFS, and CSS outcomes, revealing substantial variability among studies. Kwan et al. [14] detected similar OS (*P* = 0.695) and CSS (*P* = 0.819) between the SR and ablation groups following a propensity score-matched analysis, while Safi et al. [15] similarly found OS (*P* = 0.28) and PFS (*P* = 0.09) to be comparable in these two treatment groups, and Zemlyak et al. [16] found these two groups to exhibit similar OS (*P* > 0.05), CSS (*P* > 0.05), and PFS (*P* > 0.05) outcomes. While 2-year OS rates in the studies conducted by Kwan et al. and Safi et al. ranged from 66–85% and 62–74% in SR and ablation groups, respectively [14, 15], in other studies the survival of patients in the SR group was significantly longer than that of patients in the ablation group [11–13, 17]. These differences may be attributable to differences in sample size, tumor size, or patient selection criteria among studies. In pooled OS, PFS, and CSS analyses, survival rates were better in the SR group, and subgroup analyses further confirmed that wedge resection was associated with superior OS, PFS, and CSS outcomes relative to ablation.

The significantly prolonged survival observed following SR was primarily attributable to the significantly lower LR rate in these patients. In addition, an analysis of 100 NSCLC patients with tumors < 1 cm in diameter indicated that 5% of these patients exhibited lymph node involvement, suggesting that such involvement should still be considered even in those with mall lesions [21]. SR thus offers the additional advantage of facilitating lymph node sampling at time of surgery, enabling clinicians to more precisely stage patients and to thereby guide treatment [21].

Pooled complication rates were similar in both groups, suggesting that both SR and ablation exhibit similar safety profiles when used to treat those with stage I NSCLC. However, patients who underwent ablation experienced significantly shorter postoperative hospitalization relative to patients treated via SR, owing to the fact that ablation is a less invasive procedure not requiring the use of general anesthesia.

There were multiple limitations to this analysis. For one, the articles included in this meta-analysis were retrospective in nature, rendering them susceptible to selection bias. Additional RCTs will therefore be required to validate and expand upon these data. Secondly, certain study endpoints were associated with significant heterogeneity, and while the sources of such heterogeneity were identified when possible, additional RCTs will be essential to establish definitive research results. Third, preoperative imaging analyses were used for the evaluation of mediastinal and hilar lymph nodes in the context of RFA without any pathologic verification, potentially leading to an underestimation of accurate patient staging, thus biasing survival outcome data.

## Conclusion

In summary, SR was associated with lower LR rats and prolonged survival relative to ablation when used to treat stage I NSCLC patients.

## Data Availability

The data that support the findings of this study are available from the corresponding author upon reasonable request.

## Abbreviations

CT: Computed tomography
CSS: Cancer-specific survival
DR: Distant recurrence
LR: Local recurrence
NSCLC: Non-small-cell lung cancer
PFS: Progression-free survival
OS: Overall survival
SR: Sublobar resection
WR: Wedge resection
